# An Effective News Recommendation Method for Microblog User

**DOI:** 10.1155/2014/907515

**Published:** 2014-04-02

**Authors:** Wanrong Gu, Shoubin Dong, Zhizhao Zeng, Jinchao He

**Affiliations:** School of Computer Science and Engineering, South China University of Technology, Guangzhou 510641, China

## Abstract

Recommending news stories to users, based on their preferences, has long been a favourite domain for recommender systems research. Traditional systems strive to satisfy their user by tracing users' reading history and choosing the proper candidate news articles to recommend. However, most of news websites hardly require any user to register before reading news. Besides, the latent relations between news and microblog, the popularity of particular news, and the news organization are not addressed or solved efficiently in previous approaches. In order to solve these issues, we propose an effective personalized news recommendation method based on microblog user profile building and sub class popularity prediction, in which we propose a news organization method using hybrid classification and clustering, implement a sub class popularity prediction method, and construct user profile according to our actual situation. We had designed several experiments compared to the state-of-the-art approaches on a real world dataset, and the experimental results demonstrate that our system significantly improves the accuracy and diversity in mass text data.

## 1. Introduction

With the rapid development of Internet, more and more people prefer reading news online or by mobile phone rather than buying the newspaper. However, massive news and blogs online also bring the users information overload problem. With a large amount of news articles, a very important issue of online news services is how to help users get interesting news that match the users' preference as much as possible, which is the problem of personalized news recommendation. Microblog has become a famous network application for the past several years [1]. Therefore, how to use microblog to recommend items (i.e., news, product, or advertisement) becomes a hot research topic for website providers.

Despite some recent advances [1–4], personalized news recommendation is facing at least three problems. First, fast and real-time processing is needed for the mass news articles every day; that is, how to classify or cluster the news articles rapidly with mass data crawled by spider swarming into the system. Second, the reading context must be considered. For instance, popular news articles would likely be more attractive for the users. Third, the popularity and freshness of news is changing dramatically over time. These three problems exist in the recommender system for other items, such as movie, music, and product. However, many critical issues of news recommendation have not been solved in previous studies.

In this paper, to address the issues mentioned above, we try to solve these in news recommendation system and propose NEMAH, an effective personalized news recommendation system based on microblog user profile building and hot subclass popularity prediction. We explore intrinsic relation between user and news, through users' interest, subclass popularity factor, and freshness. In summary, the three main contributions of our paper are as follows.

- *A Novel Framework for News Partition (See Section 4)*. News classification and subclass clustering are important steps in news recommendation processing. We propose 2-stage news partition framework. First, the news articles are divided into several categories using our proposed hybrid classification method (see Section 4.2). Then, we cluster the articles in a given class into several clusters to represent news subclasses (see Section 4.3). Such representation can help news recommendation system easily build and update news database rapidly.
- *A Subclass Popularity Prediction Method for News Recommender System (See Section 5)*. Users not only like reading the news articles they are interested in, but also like the hot news, and by this phenomenon we can call the users' social preference. In general, a real-time news recommendation system is difficult to instantly obtain the statistical information of global users' attention on a specific piece of news or subclass. Therefore, we synthetically analyze the historical data crawled from web and propose a news subclass popularity prediction model based on spectral analysis of time series.
- *A Novel Application Using Microblog for User Profile Construction (See Section 6).* Microblog is the most mainstream form of grassroots media, where users can express their views and retweet the information they agreed on or are interested in. In this paper, we propose a user profile construction method based on microblog content and user behavior.

The rest of this paper is organized as follows.  Section 2 covers related work relevant to personalized news recommendation.  Section 3 describes the recommendation framework of NEMAH. Section 4 presents the classification and subclass clustering methods we design. In Section 5, the news subclass popularity prediction model will be introduced. Section 6 reports the user profile construction method we put forward and Section 7 introduces the recommending model. Extensive experimental results are reported in Section 8. Finally, Section 9 concludes this paper.

## 2. Related Work

News recommender system is an important application on recommendation and has attracted more and more attention recently. Existing news recommendation methods can be roughly divided into three categories: content-based, collaborative filtering, and hybrid methods.

*Content-Based.* This method uses the user's reading history in terms of content to recommend similar items. In the opinion of Schafer [5], he called this* Item-to-Item Correlation* method. In news recommender, generally, news article is often represented as a vector space model (VSM) or topic distributions. Reference [6] employed TF-IDF to construct VSM and utilized *K*-nearest neighbor method to recommend news to specific user. Reference [7] employed the Naïve Bayesian classifier to classify web pages and construct user profile. Liu et al. [4] (called ClickB in Experimental Evaluation section) proposed the recommendation method using news content based on click behavior. In our work, we classify news articles by VSM and express the articles with TF-IDF weight for each word. Content-based method is easy to express and implement. But it should be noted that not all data are easy to express as VSM, such as audio, image, and video news data [8]. Another problem is content similarity, for example, a user would not like to read similar news many times from news recommender using content-based method. In our work, we diversify news articles due to the distribution of crawled news articles and the preference of the given user.

*Collaborative Filtering.* This method utilizes the behaviors of user on item to recommendation. In other words, collaborative filtering method is content-free and can be roughly divided into two subcategories: heuristic-based and model-based. For the former, its recommended process is inspired by the real-world phenomena [9]. The latter one trains a model for predicting the utility of the current user *u* on item *j*, such as [10, 11] (called Goo in Experimental Evaluation section). Purchasing and rating are the most important behaviors in collaborative filtering recommendation system. But in news recommender system, the rating can be seen as binary, where a click on a piece of news can be represented as 1 rating and 0 rating otherwise [11]. The success of the collaborative filtering-based recommendation system relies on the availability of lots of users and items. But a lot of users have behaviors on only a few items. We can observe that the user-item matrix is a spare matrix that will lead to poor recommendation [12]. One way to solve this problem is by using the demographic of users to calculate the similarities between users, such as age, gender, education, area, or employment. Another approach is that which employs the behaviors through relationship among users, such as review, retweet, and favorite. In our work, we utilize the microblog information to solve the issue discussed above. 

*Hybrid Method.* This method combines collaborative filtering, content-based methods, and other factors [13]. Many news recommendation methods are hybrid, such as Bilinear [14], Bandit [15], and SCENE [3], which will be discussed and analysed in Experimental Evaluation section.

From the perspective of news recommendation, our work is similar to SCENE [3], EMM News Explorer [16], and Newsjunike [17] in the use of news content and named entities for news recommendation. However, SCENE did not consider the subclass popularity period, EMM News Explorer did not provide personalized recommender, and Newsjunike did not address the issues as we do in classification and user profile construction.

## 3. Recommendation Framework

**Figure 1:**
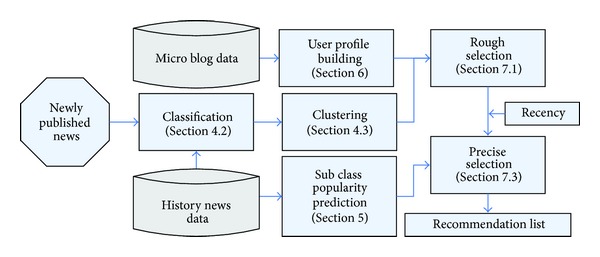
The overview of NEMAH framework.

Figure 1 shows the brief framework of our proposed system, NEMAH. This recommendation is performed by the following four modules: Classification and Clustering Module, Subclass Popularity Prediction Module, User Profile Module, and Recommendation Module. These major components and the processing flow in our framework are described as follows.

*(1) Classification and Clustering Module.* News categories on this module, customized by Press and Publication Administration of the People's Republic of China, are divided into 23 categories. As key persons (named entity of type person) play an important part on the news classification, we proposed a hybrid classification method based on the classical classification method and the key persons. A large number of experiments show that adjusting the weight of the key persons in the hybrid classification help to get a good news classification performance. After obtaining the rough classes, we cluster the subclasses using a cluster method which maximizes the average of neighborhood points. 

*(2) Subclass Popularity Prediction Module.* Different periods have different popular subclasses. People would like to focus on the popular subclasses rather than spend much time on searching and selecting information. Sometimes, even the users themselves have no idea what they want. Therefore, the subclass popularity prediction technology will help users save their time and improve their experience on using recommendation system. On the research of network news we found that some subclasses presented time period significantly. For the popular subclasses, we can assign a higher weight for recommending. In this paper, we used time series spectral analysis method to predict the popularity of subclasses. 

*(3) User Profile Module.* This module is used to extract the preference model of users. We used the microblog of users tweeted or retweeted for establishing the users profile model for representing users' interest. This procedure combines text analysis, text classification, and accessing some particular factors (i.e., key name and place name appeared in microblog). 

*(4) Personalized News Recommendation.* We use user profile and the subclass to determine the candidate subclasses firstly. And then we calculate a user's utility on news item by a greedy strategy and rank the recommended list through the popularity of news article in a special subclass and the news article's recency. Note that when recommending specific news items using our system, the class and the subclass of the news articles are utilized. Moreover, the other properties of news items, such as freshness (recency) and popularity (subclass popularity prediction), are synthesized into the final recommended ranking list as adjustment factors.

## 4. Classification and Clustering Module

**Figure 2:**
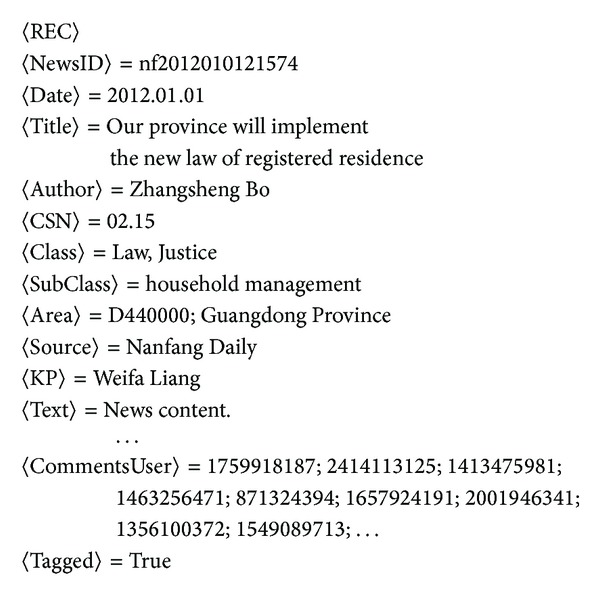
The storage structure of a piece of history news. Remark: The useful elements in this paper are: 〈NewsID〉, 〈Date〉, 〈Title〉, 〈CSN〉, 〈Area〉, 〈KP〉, 〈Text〉, 〈CommentsUser〉, in which 〈CommentsUser〉 shows the user list of whom comments this news article, 〈CSN〉 denotes the class and sub-class ID of this news article (e.g., 〈CSN〉  =  02.15 means that class ID is 02 and sub-class ID is 15), and 〈KP〉 is the named entity of type person which will be discussed in Section 4.2.

**Figure 3:**
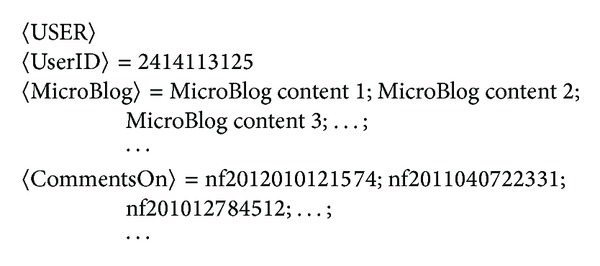
The storage structure of a user. Remark: 〈MicroBlog〉 lists the messages tweeted or retweeted by the user, 〈CommentsOn〉 denotes the news articles which are commented on by this user.

Classifying massive network news is conducive to the subsequent process on the news applications. Internet news recommendation requires response as soon as possible to show the recommended list to users. In NEMAH, given a set of news items *N* = {*n*_1_, *n*_2_,…, *n*_*M*_}, where |*N*| = *M*, our goal is to generate a classification result $\bar{C}=\{C_{1},C_{2},\ldots ,C_{K}\}$, where *K* is a predefined classification number (e.g., *K* = 23 in this paper). Class names are shown in Table 1. Besides, each class can be divided into several subclasses using our proposed clustering module, *C*_*i*_ = {*SC*_*i*1_, *SC*_*i*2_,…, *SC*_*im*_}. The storage structure of our history news and a user are shown in Figures 2 and 3, respectively.

### 4.1. Feature Selection

In the processing of text corpus, the dimension of each item will be very large (i.e., more than ten thousand in the same cases) that would need to select the main features for representing the document. Generally, there are three classical feature selection methods in text processing: Mutual Information [18], Information Gain [19], and CHI Statistics [20]. These methods are inclined to choose the rare words, which are not reliable in classification on some corpus. Therefore, in order to solve this and reduce the computational burden in the process of news articles classification, we must filter out some sporadic low-frequency words; the two concrete steps to filter are shown below.

*(1) Rough Selection Using Document Frequency of Feature Words.* In training corpus, let *t*_*i*_ be a word; we define *Df*_*i*_ as total relative document frequency, which denotes the ratio that the number of documents containing *t*_*i*_ occupies over the whole number of documents. When the *Df*_*i*_ is greater than a threshold *α*, it means that the word *t*_*i*_ is a high-frequency word in training corpus, and we add it into *Tem*_1_ set. For a given class *C*_*k*_, we define *Df*_*ik*_ as class relative document in class *C*_*k*_, which denotes the ratio that the number of documents in class *C*_*k*_ occupies over the whole number of documents in class *C*_*k*_. When the *Df*_*ik*_ is greater than a given threshold *β*, it means that *t*_*i*_ is a high-frequency word in this class, and then we add it into *Tem*_2_ set. According to our experiment and corpus, we roughly set the *α* = 0.01 and *β* = 0.1 in order to avoid the fault or omit selection. This rough selection process selects the words which appear frequently in all corpus and classes [21]. The result of rough feature selection is *Tem*′ = *Tem*_1_ ⋂ *Tem*_2_. 

*(2) Precise Selection Using Index of Discrimination between Word and Class.* We employ [22] method to define the index of discrimination between word and class as follows:

(1) $$\begin{array}{c} R\left(t_{i},C_{k}\right)=\frac{P\left(t_{i}\in C_{k}\right)}{\max_{C_{j}\neq C_{k}P(t_{i}\in C_{j})}}, \end{array}$$

where *P*(*t*_*i*_ ∈ *C*_*k*_) denotes the probability of word *t*_*i*_ in class *C*_*k*_ and max⁡_*C*_*j*_≠*C*_*k*_*P*(*t*_*i*_∈*C*_*j*_)_ denotes the maximum probability of word *t*_*i*_ in other classes except *C*_*k*_. The *P*(*t*_*i*_ ∈ *C*_*k*_) can be represented as follows:

(2) $$\begin{array}{c} P\left(t_{i}\in C_{k}\right)=\frac{tf\left(t_{i}\in C_{k}\right)+1}{\sum_{t^{\prime }}tf\left(t^{\prime }\in C_{k}\right)+1}, \end{array}$$

where *tf*(*t*_*i*_ ∈ *C*_*k*_) denotes the frequency of *t*_*i*_ appearing in class *C*_*k*_, *t*′ denotes the word different to *t*_*i*_ from *Tem*′, and ∑_*t*′_ *tf*(*t*′ ∈ *C*_*k*_) denotes the sum frequency of *t*′ appearing in class *C*_*k*_. The *t*_*i*_ is the representative word in class *C*_*k*_ when the index of discrimination *R*(*t*_*i*_, *C*_*k*_) is greater than a threshold *γ*. We can use selection proportion threshold *T* to decide parameter *γ*, which will be discussed in our Experimental Evaluation section later. We can obtain the representative words set when the process above is done for each class. Rough selection step can save calculation time that is used to exclude the words which are certainly not the feature words.

### 4.2. Classifying News Items

In real Internet world, classification or clustering on massive news data requires lots of computational power. To tackle this issue on news recommendation, we employ One Versus All method [23] (One Versus All is a two-class classification method) and consider the key persons on news articles. In this paper, news article classification is considered as a plurality of two-class classification problem. For a class *C*_*k*_, if document *d*_*i*_ belongs to class *C*_*k*_, it is tagged by 1 for class *C*_*k*_ as a positive sample and tagged by −1 as a negative sample otherwise. This method is to construct the projective vector *p*_*k*_ between text matrix *A* and class vector *y*, and we employ the ridge regression method [24] shown in the following:

(3) $$\begin{array}{c} C=\text{argmi}{\text{n}}_{p_{k}}{||y-p_{k}^{T}A||}^{2}+\theta {||p_{k}||}^{2}, \end{array}$$

where *θ* is a positive parameter used to adjust the estimation error. To solve the minimization problem above, we should find the partial derivative of *p*_*k*_ and set the partial derivative to 0, and then we can obtain the equation shown below:

(4) $$\begin{array}{c} p_{k}={\left(AA^{-1}+\theta I\right)}^{-1}Ay^{T}, \end{array}$$

where *I* is a unitary matrix with the same dimension of *A*. Because the training set is divided into *K* categories, we can obtain a group of projective vectors: *P* = {*p*_1_, *p*_2_,…, *p*_*K*_}. We utilize code matrix *M* to describe the correlation between different classes got from two-class classification. Assuming that class *C*_*k*_ has *N*_*k*_ trained documents *D* ^*k*,*j*^, where *j* ∈ [1, *N*_*k*_], the element of *M* which denotes the correlation between two classes can be calculated by

(5) $$\begin{array}{c} M_{KK^{\prime }}=\frac{1}{N_{k}}\overset{N_{k}}{\underset{j=1}{\sum }}\mathrm{sgn}\left(\langle p_{k^{\prime }},D^{k,j}\rangle \right), \end{array}$$

where *p*_*k*′_ denotes the projective vector of *C*_*k*′_. If 〈*p*_*k*′_, *D* ^*k*,*j*^〉 is greater than 0, the return value of function sgn⁡ is 1 and otherwise 0. When new articles come, the similarity between article and class can be calculated by the following equation:

(6) $$\begin{array}{c} \text{Sim}\left(B,C_{K}\right)=\overset{k}{\underset{K^{\prime }=1}{\sum }}M_{kk^{\prime }}Q_{k^{\prime }}=\overset{k}{\underset{K^{\prime }=1}{\sum }}M_{kk^{\prime }}\mathrm{sgn}\left(\langle p_{k^{\prime }},D^{k,j}\rangle \right), \end{array}$$

where *B* denotes a new article. At last, we can obtain the class of *B* through the maximum of function Sim(·, ·):

(7) $$\begin{array}{c} C\left(B\right)=\arg\max_{C_{k}}\text{Sim}\left(B,C_{k}\right). \end{array}$$

In order to further improve the classification accuracy and utilize the manual labor rationally, we propose a method with considering key person (named entity of type person) to improve the ability of classification when key persons appear, as shown in the following:

(8) P(Ci ∣ B)=(1−α)Sim(B,CK)∑i=1KSim(B,Ci)+αP(Ci ∣ Bk)P(Bk ∣ dj),

where Sim(*B*, *C*_*K*_)/∑_*i*=1_^*K*^Sim(*B*, *C*_*i*_) denotes the probability score of article *B* on class *C*_*k*_ obtained by the method we mentioned above, *B*_*k*_ denotes the key person that appeared in the article *B*, and *P*(*B*_*k*_ | *B*) = 1 when *B*_*k*_ appeared in *B*; otherwise, *P*(*B*_*k*_ | *B*) = 0. In other words, if a new article has not appeared in any key person, we could not implement the key person factor on it. *α* is the balance parameter on these two methods. The *P*(*C*_*i*_ | *B*_*k*_) is computed as

(9) $$\begin{array}{c} P\left(C_{i}\;|\;B_{k}\right)=\frac{P\left(C_{i}\right)p\left(B_{k}\;|\;C_{i}\right)}{\sum_{i=1}^{M}P\left(C_{i}\right)p\left(B_{k}\;|\;C_{i}\right)}. \end{array}$$

### 4.3. News Subclass Clustering

After obtaining the rough classification results, we need to separate every news class into subclass *SC*_*ix*_. A natural way to detect subclasses of an Internet text corpus is typically done using clusterings, for instance, such as *K*-means or hierarchical clusterings. In NEMAH, we propose a subclass clustering method to obtain subclasses. Each subclass is represented as a subclass vector *T* = {〈*t*_1_, *w*_1_〉, 〈*t*_2_, *w*_2_〉,…}, where *t*_*i*_ and *w*_*i*_ denote the representative word and its corresponding weight, respectively. We call this cluster method as Maximizing Neighborhood method because of the main idea of algorithm.

*(1) Solving Subspace Projection Problem by Maximizing the Average of Neighborhood.* For each document *x*_*i*_ in a text space *X* ^0^, the neighbor documents can be divided into two subsets according to the distance to the *x*_*i*_: similar neighborhood set Θ_*i*_^*o*^ and heterogeneous neighborhood set Θ_*i*_^*e*^, where Θ_*i*_^*o*^ contains the top *ξ* nearest neighbors which belong to the same class of *x*_*i*_ and Θ_*i*_^*e*^ contains the top *ζ* nearest neighbors which do not belong to the same class of *x*_*i*_. In the text corpus, all data points' average distance out of class and within class can be expressed as follows:

(10) $$\begin{array}{c} P_{i}=\sum_{x_{p}\in \Theta_{i}^{e}}\frac{{||x_{i}-x_{p}||}^{2}}{\left|\Theta_{i}^{e}\right|},\\ Q_{i}=\sum_{x_{q}\in \Theta_{i}^{o}}\frac{{||x_{i}-x_{q}||}^{2}}{\left|\Theta_{i}^{o}\right|}. \end{array}$$

All data points in the text corpus average out of class distance and the average within-class distance expression are as follows:

(11) $$\begin{array}{c} P=\sum_{i}P_{i}=\sum_{i}\sum_{x_{p}\in \Theta_{i}^{e}}\frac{{||x_{i}-x_{p}||}^{2}}{\left|\Theta_{i}^{e}\right|},\\ Q=\sum_{i}Q_{i}=\sum_{i}\sum_{x_{q}\in \Theta_{i}^{o}}\frac{{||x_{i}-x_{q}||}^{2}}{\left|\Theta_{i}^{o}\right|}. \end{array}$$

The subclass clustering problem can be considered as a projection of text space to a subspace. For instance, let *y*_*i*_ be a projection space of *x*_*i*_ after projecting; we can express *y*_*i*_ = *W* ^*T*^ *x*_*i*_. The principle of this projection is maximizing the average distance of different classes and minimizing the average distance within each class [25], as shown in the following:

(12) r=∑i(∑xp∈Θie||xi−xp||2|Θie|−∑xq∈Θio||xi−xq||2|Θio|)=tr[WT(∑i∑xp∈Θie||xi−xp||2|Θie|−∑i∑xq∈Θio||xi−xq||2|Θio|)W]=tr[WT(P−Q)W],

where *tr*(·) denotes the trace of a matrix and the constraint of this equation is *W* ^*T*^ *W* = *I*. And then maximize the equation shown as follows:

(13) $$\begin{array}{c} \max\left\{tr\left[W^{T}\left(P-Q\right)W\right]\right\}. \end{array}$$

*(2) The Quick Affinity Propagation Clustering on Subspace.* After projecting the initial text vector space into subspace through projective matrix, it can generate *K* clusters with employing *K*-Affinity Propagation (*K*-AP) (this method will be more suitable for text clustering because it can achieve more reasonable clusters than the traditional clustering methods [26]) implemented in subspace. Let the similarity of *y*_*i*_ and *y*_*j*_ in subspace *Y* = {*y*1, *y*2,…, *y*_*n*_} be *S* = {*s*_*ij*_}; the target of *K*-AP is to find the *K* real samples *E* = {*e*_1_, *e*_2_,…, *e*_*K*_}, which denotes the *K* classes *C* = {*C*_1_, *C*_2_,…, *C*_*K*_}. And then maximize the following objective function:

(14) $$\begin{array}{c} \max F\left({\left\{C_{j}\right\}}_{j=1}^{K}\right)=\overset{K}{\underset{j=1}{\sum }}\sum_{y_{i}\in C_{j}}s\left(y_{i},e_{j}\right), \end{array}$$

where *e*_*j*_ belongs to *C*_*j*_. The objective function can be transformed into 0-1 integer programming problem when introducing the binary parameter *B* = {*b*_*ij*_ ∈ {0,1}, *i*, *j* = 1,…, *n*}, as shown in the following:

(15) $$\begin{array}{c} \max F\left(\left\{b_{ij}\right\}\right)=\overset{n}{\underset{i=1}{\sum }}\overset{n}{\underset{j=1}{\sum }}b_{ij}s\left(y_{i},y_{j}\right). \end{array}$$

Equation (15) has three constraints: (1)  *b*_*ii*_, if *b*_*ji*_ = 1, (2)  ∑_*j*=1_^*n*^ *b*_*ij*_ = 1, and (3)  ∑_*i*=1_^*n*^ *b*_*ii*_ = *K*, where *b*_*ij*_ = 1 when *y*_*i*_ considers *y*_*j*_ as a sample and *b*_*ii*_ = 1 when *y*_*i*_ is a sample itself. For the first constraint, *y*_*i*_ is a sample when *y*_*j*_ considers *y*_*i*_ as a sample. For the second one, it means that each data point has only one sample point. For the last one, it means that the number of samples is *K*, which can ensure that *K*-AP method generates *K* clusters.

*(3) Hybrid Learning of Subspace Projection and Clustering on Adaptive Subspace.* The class information updated on subspace clustering process can be utilized as a priori knowledge in the next processing on subspace projection, and after several iterations until convergence we can obtain the global optimal clustering result. The iteration processing is as follows:

 
* X* ^0^ → *K*-AP → *L* ^0^  →  SubSpace  →  *W*_1_, Score_1_
 
* Y* ^1^ = *W*_1_^*T*^ *X* ^0^ → *K*-AP → *L* ^1^  → SubSpace →  *W*_2_, Score_1_
 
* *⋯→⋯→⋯→⋯→⋯
 
* Y* ^*t*^ = *W*_*T*_^*T*^ *X* ^0^ → *K*-AP → *L* ^*t*^  → SubSpace →  *W*_*t*+1_, Score_*t*+1_.

It must compute the convergence function value in each iteration:

(16) $$\begin{array}{c} {\text{Score}}_{t+1}=tr\left[W_{t+1}^{T}\left(P\left(L^{t}\right)-Q\left(L^{t}\right)\right)W_{t+1}\right], \end{array}$$

where *P*(*L* ^*t*^) and *Q*(*L* ^*t*^) denote the average distances between classes and within class which are calculated by (11) according to the class vested instruction matrix *L* ^*t*^. The iteration will be finished when it meets the condition of convergence: Score_*t*+1_ − Score_*t*_ ≤ *ϵ* or reaches the max. number of iterations. The parameters of our clustering method are the number of points *η* which are the nearest in class and the number of points *ζ* which are the nearest out of class. We did cross-fold validation to train these parameters and we found that selecting *ζ* = *η* = 13 for all classes per 1,000 documents would perform better.

*Discussion.* The motivation of this module (classification and clustering) is to find the user's preference (subclass level) and track the hotness of a newly published news in a given subclass.

## 5. Subclass Popularity Prediction Module

On the explosion of information today, the fast pace of life makes people focus their attention on the popular subclass rather than spend much time searching and selecting information. Sometimes, even users themselves have no idea what they really want. Therefore, the hot subclass prediction technology with recommendation function has become very important. News subclass popularity prediction can improve the performance of news recommender system. Besides, it can also improve the display function of popular news modules on website automatically, reduce the workload of website editors, and improve the users' browsing experience.

**Figure 4:**
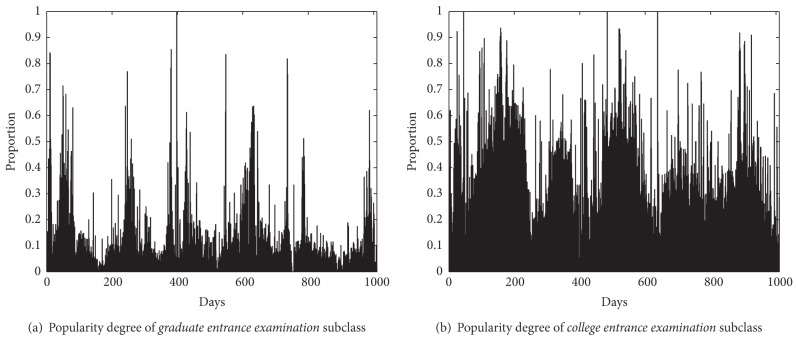
Periodic subclass news distribution.* Remark.* *x-*axis denotes the date from August 1, 2009, to May 3, 2012; *y*-axis denotes the value of *H*_*k*_ in (17).

On the study of historical statistical data on news subclasses, we found that some subclasses are popular periodically. For instance, the subclass* college entrance examination* will appear highly popular about June every year in China, and a lot of news articles and comments focus on this subclass at that time, as shown in Figures 4(b) and 4(a) that show the data of college entrance examination subclass. In this paper, we define the news subclass' degree of concern according to the number of news articles and their comments, as shown in the following:

(17) Hk=λHne(k)+(1−λ)Hre(k)=Nre(k)Nne+NreNne(k)Nne+Nne(k)Nne+NreNre(k)Nre,

where *H*_*ne*_^(*k*)^ denotes the popular degree of news article on the *k*th subclass, *H*_*re*_^(*k*)^ denotes the popular degree of comment on the *k*th subclass, *λ* is a weight of popular degree of news article and the value is *N*_*re*_^(*k*)^/(*N*_*ne*_ + *N*_*re*_), *N*_*re*_^(*k*)^ denotes the number of reviews on the *k*th subclass, *N*_*re*_ denotes the number of reviews on all corpus, *N*_*ne*_^(*k*)^ denotes the number of news articles on the *k*th subclass, and *N*_*ne*_ denotes the number of news articles on all corpus. According to the experiments of time series analysis on our corpus, we found that most subclasses are suitable for implementing* spectral analysis* method [27].

Any stationary sequence modeling can be extended to many cosine waves with different frequencies, amplitude, and phase combination. This analysis method is called* time domain based analysis method*. The linear combination of *m* cosines with arbitrary amplitudes, frequencies, and phases; it is shown in the following:

(18) $$\begin{array}{c} Y_{t}=A_{0}+\overset{m}{\underset{j=1}{\sum }}\left[A_{j}\cos\left(2\pi f_{i}t\right)+B_{j}\sin\left(2\pi f_{i}t\right)\right]. \end{array}$$

It can get the values of *A* and *B* by ordinary least squares fitting regression. When the frequency is a special form, the calculation will become very simple. If *n* is an odd number, which can be expressed as *n* = 2*k* + 1, then the frequency with the form of 1/*n*, 2/*n*,…, *k*/*n* is called Fourier frequency. The estimated parameters are as follows:

(19) A^0=Y−,A^j=2n∑t−1nYtcos⁡(2πtjn),B^j=2n∑t−1nYtcos⁡(2πtjn).

If the sample size is even, which can be expressed as *n* = 2*k*, (19) still holds. But the equation will change to the following when *f*_*k*_ = *k*/*n* = 1/2:

(20) $$\begin{array}{c} \hat{A}{}_{k}=\frac{1}{n}\overset{n}{\underset{t=1}{\sum }}{\left(-1\right)}^{t}Y_{t},\;\;\hat{B}{}_{k}=0. \end{array}$$

**Definition 1**

When the sample size is odd, namely, *n* = 2*k* + 1, we define the cycle diagram whose frequency *f* = *j*/*n* (*j* = 1,2,…, *K*) as *I*, as shown in the following equation:

(21) $$\begin{array}{c} I\left(\frac{j}{n}\right)=\frac{n}{2}\left(\hat{A}{}_{j}^{2}+\hat{B}{}_{j}^{2}\right). \end{array}$$

If the sample size is even, (19) still can get the $\hat{A}$ and $\hat{B}$ values, and the cycle diagram is the same as the odd case. But in the extreme frequency case, for example, when *f* = *k*/*n* = 1/2, the cycle diagram is shown in the following equation:

(22) $$\begin{array}{c} I\left(\frac{j}{n}\right)=n{\left(\hat{A}{}_{j}\right)}^{2}. \end{array}$$

**Figure 5:**
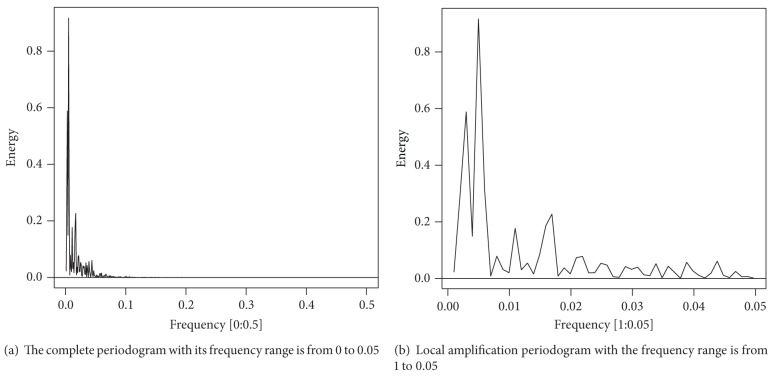
Periodogram of the popularity degree of* college entrance examination* subclass.* Remark*. *x*-axis denotes the possible frequency of the popularity degree; *y*-axis represents the energy of the corresponding frequency.

The periodogram with frequency *f* = *j*/*n* is inversely proportional to the square value of the corresponding regression coefficients. Therefore, the peaks of periodogram show the relative intensity of sine-cosine pairs in different frequencies, as shown in Figure 5.

In Figure 5, the periodogram has two peaks: 0.004970179 and 0.002982107; namely, the subcycle *T* = 1/*f* may be 201 days and 335 days. The other peaks are too low that they can be neglected. The two frequencies are selected for building the model, which means that the model has two pairs of sine-cosine in it, as shown in the following:

(23) Yt=β+β1cos⁡(2πf1t)+β2sin(2πf1t) +β3cos⁡(2πf2t)+β4sin(2πf2t)+et.

Using spectral analysis method for prediction has several steps. First, we should use the periodogram for getting the value and number of strong frequencies. Second, model is generated by the value and number of strong frequencies. Finally, we predict future data values according to the model which only requires a time parameter.

*Discussion.* The motivation of this module is to obtain the hotness of each subclass. Some new studies also take into account the popularity of the newly published news article. For example, SCENE [3] used the popular degree which is computed as the ratio of the number of users accessing the article. However, for the newest popular news article *n*_*i*_, its clicked number would be less than the news article published several hours or days before.

## 6. User Profile Module

In order to capture a user's reading interest on news items, generally, personalized news recommendation system needs to construct the user's profile. Traditionally, the user profile can be captured by the track of user reading history. A survey of various user profile construction techniques is provided in [28, 29]. In this paper, we use the microblog to construct the user's profile. The reason is that the user who is interested in some subclasses will tend to tweet or retweet microblog on these subclasses. For instance, a user tweets or retweets many messages about basketball game that we can deduce that this user may like reading basketball news reports (i.e., NBA, CBA, etc.). Besides, many readers tend to glance at news articles and are interested in some key persons' names. Moreover, people from different areas would tend to read the news from their living city or their hometown. Based on the above analysis, we propose to construct users' profiles by the exploration on the four factors discussed above: microblog content, place name, and preferred key persons. In order to reduce the computational complexity, preference is also taken into account in our model that can be represented by a vector *U*_*pf*_ = {*τ*, *ρ*, *κ*}. Consider the following.

1. *τ* represents the key index words distribution of microblogs which user tweeted or retweeted in the past, and it can be expressed as a vector {〈*t*_1_, *w*_1_〉, 〈*t*_2_, *w*_2_〉,…}, where each element consists an index word and its corresponding weight.
2. *ρ* represents the place names which appeared in the microblog of a specific user, and it can be expressed as {〈*p*_1_, *w*_1_〉, 〈*p*_2_, *w*_2_〉,…, 〈*p*_*i*_, *w*_*i*_〉,…}, where *p*_*i*_ denotes a place name and *w*_*i*_ denotes the number of this place appearing in the tweets of the given user. We collect all the cities and provinces names in China. Some place names are subordinate to others; for instance, GuangZhou city is subordinate to GuangDong province. In this case, system will add weight to GuangDong using *w*_GuangDong_ + = *w*_GuangZhou_ when GuangZhou appears.
3. *κ* represents the list of key persons' name extracted from the users' microblog: {〈*k*_1_, *w*_1_〉, 〈*k*_2_, *w*_2_〉,…}, where the name list is constructed from* NanFang Daily* training corpus which the key persons' names have tagged in each news article.

## 7. Personalized News Recommendation Module

The recommendation module can be divided into two steps:* Rough Selection* (see Section 7.1) and* Precise Selection* (see Section 7.3). For the first step, some subclasses are matched due to the user's preference. And then we select the news articles from these subclasses by our selection strategy.

### 7.1. Rough Selection: Subclass Selection for a User

Once we obtain the subclasses and user's profile, we can calculate the similarity between each subclass and a given user. We can use TF-IDF weight to represent the vector of a given subtopic *τ*_*s*_ = {〈*t*_1_, *w*_1_〉, 〈*t*_2_, *w*_2_〉,…}. The similarity between a subclass and a user (represented as *τ*_*u*_ = {〈*t*_1_, *w*_1_〉, 〈*t*_2_, *w*_2_〉,…} in *U*_*pf*_; see Section 6) is computed by cosine similarity. In general, users tend to have their preference on some special subclasses; that is, they are not interested in all subclasses. Therefore, we can roughly select some subclasses with a similarity threshold. This threshold is set to be equal to the 30% of all similarity scores ranking with respect to a given user.

### 7.2. News Profile Construction

After obtaining news clusters that user might be interested in, the next step is to select specific news articles to the given user. Similar to user, we initially maintain a news profile for each news article and then model the recommendation as a budgeted maximum coverage problem and solve it by a greedy selection algorithm. A news profile contains many similar factors, for example, key person, place, clustering of belonging, recency, popularity, and so forth. For the popularity, as we discussed above, we used *H*_*k*_ to represent the popularity degree of *k* cluster. For the recency, the score is represented as the following:

(24) $$\begin{array}{c} \text{Rec}\left(i\right)=\frac{i_{c}-i_{p}}{24*60}, \end{array}$$

where Rec(*i*) function returns the recency score of news article *i*, and *i*_*c*_ and *i*_*p*_ denote the current time and published time, respectively.

In this paper, news profiles are helpful to evaluate how the news article can satisfy the user. Given a news profile *N*_*pf*_ = {*ρ*, *κ*, *υ*} and a user's profile *U*_*pf*_ = {*ρ*, *κ*, *υ*}, the similarity between *N*_*pf*_ and *U*_*pf*_ is computed as

(25) sim(Npf,Upf)=γ1sim(ρn,ρu)+γ2sim(κn,κu)+γ3sim(υn,υu),

where *γ*_1_, *γ*_2_, and *γ*_3_ are parameters to control how we trust or weigh the corresponding components and are set to 1 in our system. Each component is calculated by the cosine similarity.

Let *E* be a finite set and *f* a real valued nondecreasing function defined on the subsets of *E* that satisfies

(26) $$\begin{array}{c} f\left(T\cup \left\{\varsigma \right\}\right)-f\left(T\right)\leq f\left(S\cup \left\{\varsigma \right\}\right)-f\left(S\right), \end{array}$$

where *S*⊆*T*, *S* and *T* are two subsets of *E*, and *ς* ∈ *E*∖*T*. Such a function *f* is called a submodular function [30]. By adding an element to a larger set *T*, the value increment of *f* cannot larger than that add an element to a smaller set *S*. This budgeted maximum coverage problem can be described as follows: given a set of elements *E* in which each element is associated with an influence and a cost defined over a domain of these elements and a budget *B*, the goal is to find out a subset of *E* which contains the largest influence while the total cost does not exceed budget *B*. This problem is NP-hard [31]. However, [31] proposed a greedy algorithm which sequentially picks up the element that increases the largest possible influence within the cost limit. Submodularity resides in each pick up step. Due to the result of [32], submodular functions are closed under nonnegative linear combinations.

### 7.3. Precise Selection: News Selection for Recommendation

In a given news subclass, we observe that most of news concentrate on similar topic, with minor difference on major aspects of the corresponding topic. Typically, a reader is interested in some aspects of the given subclass, but not all of them. Based on this intuition, our news selection strategy can be described as follows.

Assuming that *𝒞* denotes the newly published news set, *𝒮* represents the selected news set and *ς* denotes the news article being selected. After selecting a piece of news *ς*, we must insure that

- the topic diversity should not deviate much in *𝒮*,
- *𝒮* should give more satisfaction to the given user,
- *𝒮* should be similar to the general topic in *𝒞*∖*𝒮*.

For each of the above strategies, similar to [3], we define a quality function *q*(*𝒮*) to evaluate the value of current selected news set *𝒮* as follows:

(27) q(𝒮)=1(|𝒮|2)∑N1,n2∈𝒮n1≠n2−sim(n1,n2)+1|𝒮|∑n1∈𝒮sim(u,n1) +1|𝒞∖𝒮|·|𝒮|∑n1∈𝒞∖𝒮∑n2∈𝒮sim(n1,n2),

where *n*_1_ and *n*_2_ denote news items, *u* denotes the given user, and sim(·, ·) function returns the similarity of its two parameters. Equation (27) contains three components corresponding to the news selection strategy we list above. *q*(*𝒮*) balances the contribution of different components. Suppose *ς* is the candidate news document; the quality increase can be represented as

(28) $$\begin{array}{c} I\left(\varsigma \right)=q\left(𝒮\cup \varsigma \right)-q\left(𝒮\right). \end{array}$$

The goal is to select a list of recommended news documents which provide the largest possible values within the budget (i.e., the budget can be regarded as the maximum number of the articles in recommended list). We can obtain a list of news documents for each subclass by adopting the greedy selection algorithm. Taking into account the other characteristics of news documents, for example, the popularity and the recency, the ranking of the selected news articles needs to be adjusted in order to make the recommended results more reasonable. Formally, given a news article *n*, the popularity and the recency can be combined as

(29) $$\begin{array}{c} n_{\phi }=\frac{H_{k_{n}}-H_{\min}}{H_{\max}-H_{\min}}-\frac{\text{Rec}\left(n\right)-{\text{Rec}}_{\min}}{{\text{Rec}}_{\max}-{\text{Rec}}_{\min}}, \end{array}$$

where *H*_*k*_*n*__ denotes the popularity degree of the subclass which the news *n* belongs to and Rec(*n*) can be obtained from (24). From the equation above, we note that the smaller the recency is, the higher the article is ranking. Besides, the greater the popularity is, the higher the article is ranking. After computing the *n*_*ϕ*_ value of the list of recommended articles, we implement a quicksort algorithm on these articles according to the *n*_*ϕ*_. By such adjustment, the generated ranking can emphasize more popular and freshness, as well as concentrate on news documents that satisfy the user's preference.

## 8. Experimental Evaluation

In this section, we provide a comprehensive experimental evaluation to show the efficacy of our proposed news recommendation system. We start with an introduction to a real-world collection obtained from a news and microblog service website, SINA. After that, we will describe the experimental design and show the results based on the recommendation framework introduced in this paper.

### 8.1. Real-World Data Set

For experiments, we gather the news data from SINA (http://news.sina.com.cn/), where the data collection ranges from August 1, 2009, to August 31, 2012. We also gather the users who comment on the these articles and their microblog from SINA (http://weibo.com/) and preprocess the data by removing microblog messages that are too short (i.e., less than 3 words) and the nonactive users (i.e., the users who tweeted or retweeted less than 10 messages) for verifying our recommendation performance. After preprocessing, 5,127 users are stored with 124,301 messages and 280,737 news articles.

### 8.2. Experiments

Our system has four major components: (1) a module responsible for classification and clustering news articles; (2) a component of constructing and updating profiles of users; (3) hot news subclass prediction based on time-series analysis; and (4) a recommendation component using news cluster and user profile accompanied by subclass popularity factor and recency. From the experimental perspective, we verify our components firstly. And then we verify our system compared to the state-of-the-art approaches and design a user study.

#### 8.2.1. Classification and Clustering Evaluation

In order to evaluate the performance of classification and clustering component, we design two experiments.

**Figure 6:**
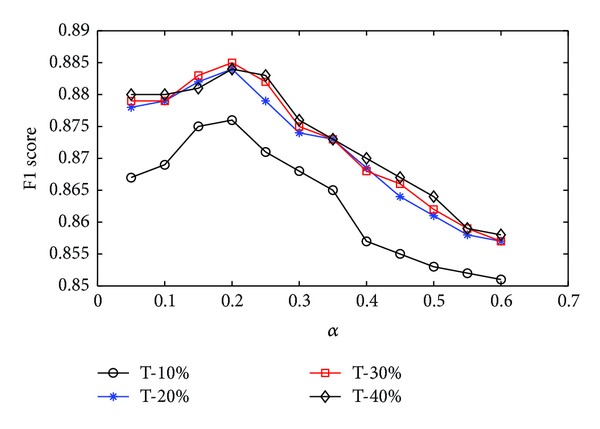
*α* parameter selection via classification.* Remark*. *y*-axis is the F1 measure score of our classification using different *α*.

*(1) Classification Comparison.* There are many classified methods in the past decade in the field of text processing. We implement the three following classification methods: the method of Cheng et al. [33], the method of Guo et al. [34], and the Naïve Bayesian (NB) method. Cheng proposed a text classification based on refining concept index and Guo employed genetic algorithm for classifying. Before using classification module, we must set the *α* in (8) and decide the threshold of feature selection through an offline experiment, as shown in Figure 6, where T-10% denotes that threshold = 10% in feature selection and F-score is Micro-F1. The performance achieves the best roughly when *α* = 0.2. From the result, we also observe that the thresholds we selected as 20%, 30%, and 40% produce similar results, so we use *T* = 20% in our processing.

Table 2 lists the recommendation evaluation results from different classifications. Based on the comparison, we know that our proposed method outperforms the classical method Naïve Bayesian and Cheng and Guo methods in terms of F1 measure. A straightforward explanation for the improvement is that our method uses less features selected by the method we proposed to represent news articles and implement a series of two-class classification to improve the similarity problem of different classes, and the most important reason may be that we implement the key persons which are classified manually into the method.

**Figure 7:**
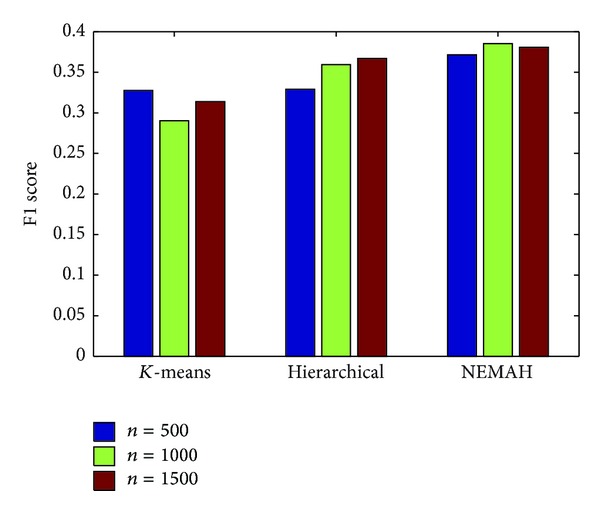
Recommendation performance of different data scales for different clustering based systems.* Remark*. *n* denotes the number of news articles for clustering.

*(2) Clustering Comparison.* In reality, we need to cluster the news articles into subclasses every day, even every hour. For our spider software, we know that more than thousands of news articles arrive per day. *K*-means and hierarchical clustering methods are the most common clustering algorithms. In order to verify our proposed method, we design the experiment as follows: (1) use 500, 1000, and 1500 as the number of newly published articles for processing; (2) for each scale of dataset, implement classification on these data; (3) perform *K*-means, hierarchical clustering, and our proposed clustering method on these data; (4) perform Top@30 news recommendation; and (5) compute the F1 score for different clustering based systems. The comparison of recommendation on different subclass clustering methods is shown as in Figure 7.

From the experimental result, we have the following observations. (1) NEMAH performs a better result compared to the other methods in terms of F1 score. (2) NEMAH is more stable than the other methods. A straightforward explanation might be that *K*-means clustering needs an initial clustering center for each cluster. Besides, with fewer parameters, our proposed method has stronger generalization and learning ability without requiring the size and distribution of text corpus.

#### 8.2.2. User Profile and Subclass Popularity Prediction Evaluation

**Figure 8:**
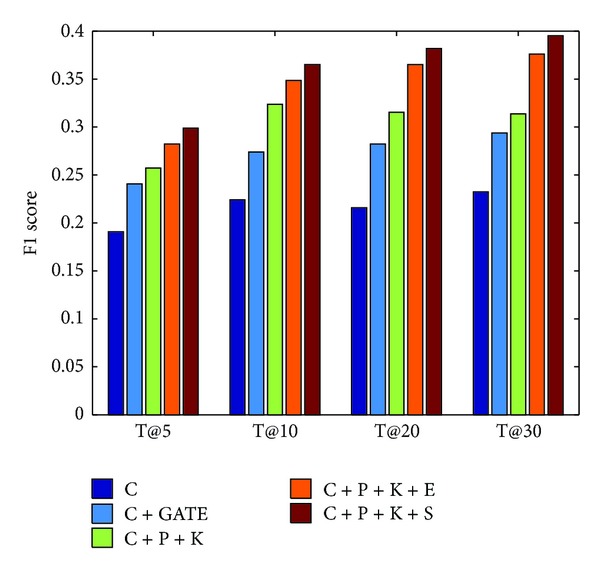
Recommendation F1 score of different profile factors and subclass popularity prediction methods.* Remark.* C: content, P: place name, K: key person name, GATE: entities name using GATE tool, E: popularity prediction using three-time exponential smoothing, and S: popularity prediction using spectral analysis (employed by NEMAH).

User profile is an important factor in a recommendation system that can affect the recommendation result significantly. Our user profile construction includes the following factors:* content*,* place name*, and* key person*. Prior approaches often use the content or similar access pattern to construct the user profile. SCENE [3] used the content, similar access pattern, and entities which are extracted by GATE [35]. In reality, the entities such as place names and key person names are stable for a period relatively.  Figure 8 shows the results of using different user profile building methods and subclass popularity prediction methods.

From this result, we observe the following. (1) Our method performs better performance than using GATE. (2) Recommendation using content only cannot perform well because microblog has not had a lot of content in its messages. (3) The Spectral Analysis employed in subclass popularity prediction can be better than the* Three-Time Exponential Smoothing* method. Although the average performance of Spectral Analysis is better than Three-Time Exponential Smoothing; in our other work about time series analysis, we found that some subclasses' cycle diagrams have less strong signal of frequencies which would tend to overfitting with a large number of sine-cosine pairs and obtain worse results in these subclasses. SCENE [3] also used the popular degree which is computed as the ratio of the number of users accessing the article and the size of the users' pool. However, this method is contradicting to the freshness. The straightforward reason is that, the freshest news may get few of clicked.

#### 8.2.3. Diversity Evaluation

The recommendation news list of NEMAH performs a great diversity on both class and subclass aspects. Let *R*(*u*) be a news recommended list of a user *u*, and the diversity of *u* can be defined as follows:

(30) $$\begin{array}{c} \text{Diversity}=1-\frac{\sum_{i,j\in R(u),i\neq j}\text{sim}\left(i,j\right)}{\left(1/2\right)\left|R\left(u\right)\right|\left(\left|R\left(u\right)\right|-1\right)}, \end{array}$$

where *i* and *j* are two different news articles in recommendation list for user *u* and sim(*i*, *j*) denotes the news profile similarity between the news item *i* and *j*. For this metric evaluation, we choose Goo [11] (a collaborative filtering based method), ClickB [4] (a content-based method), Bilinear [14], Bandit [15], and SCENE [3] (a hybrid method using LSH for clustering and greedy algorithm for news selection) as the comparison baselines.  Table 3 shows the result of the diversity result with |*R*(*u*)| = 10, |*R*(*u*)| = 20, and |*R*(*u*)| = 30 in which we use *T*@10 to represent |*R*(*u*)| = 10.

From Table 3, we can see that our system outperforms the others significantly, and the straightforward reason is that we diverse the news not only according to the preference of user but also according to the distribution of candidate news articles. With the recommendation list enlarged, the diversity decreases significantly on the baseline methods because they rely on the preference of user too much.

#### 8.2.4. System Accuracy Evaluation

**Figure 9:**
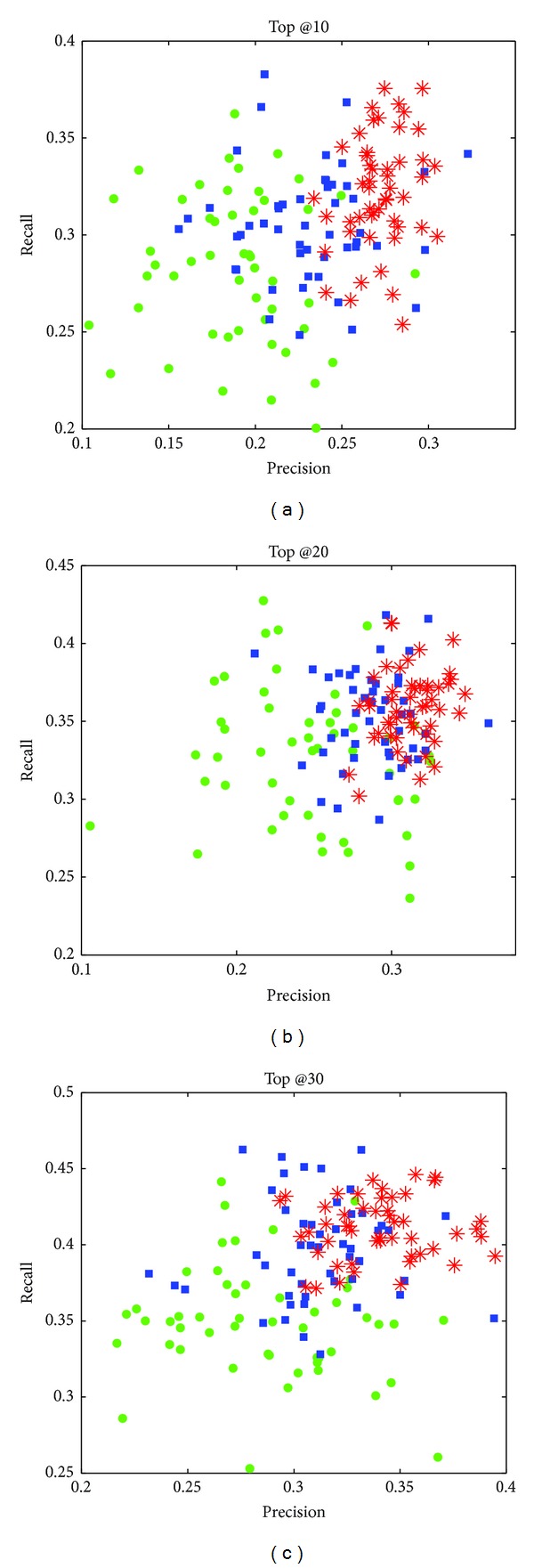
Precision-recall plot of different recommendations.* Remark.* ∘ (green) denotes the bandit-based recommender; □ (blue) denotes the SCENE recommender; and ∗ (red) represents NEMAH.

In order to verify the effectiveness of our proposed NEMAH, we implement a recommender system that models the recommendation as a contextual bandit problem [15]. Also, we implement the SCENE [3] prototype system which employed LSH (Locality Sensitive Hashing) for news clustering and used greedy selection for user recommendation. For each method, we select 50 users to provide news recommendation results for them.  Figure 9 shows the comparison results as Top @10, Top @20, and Top @30 news items for each user.

In the above experiments, we can observe that, besides the higher accuracy, the distribution of our system is more stable than other approaches.

**Figure 10:**
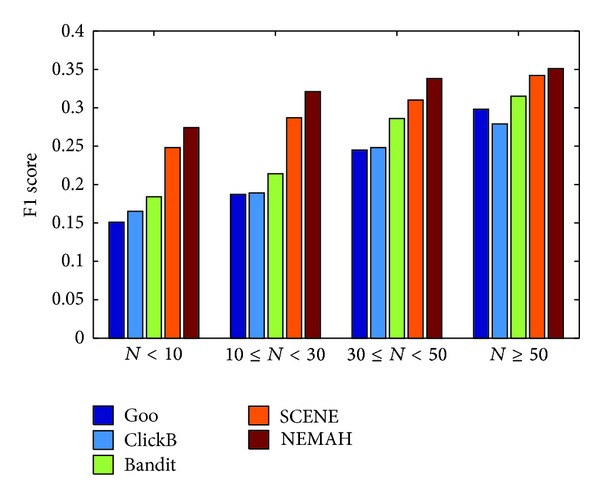
Comparison of F1 score of different approaches for different user groups.* Remark*. *N* denotes the number of news articles per day which is read by a user.

In reality, if users read a few of news articles every day, many news recommendation systems could not outperform good result for these users. Our system can address this problem due to the microblog user profile building.  Figure 10 shows the comparison results for different users groups for all users (5,127 users). Suppose a reader reads *N* news articles per day. From this figure, we can know that our proposed system can outperform a reasonable result when it is subject to* nonactive* users. SCENE also outperforms not bad result. The reason is that NEMAH and SCENE consider the named entities refereed by users. Besides, NEMAH takes into account the popular degree on a news article.

#### 8.2.5. A User Study on NEMAH

**Figure 11:**
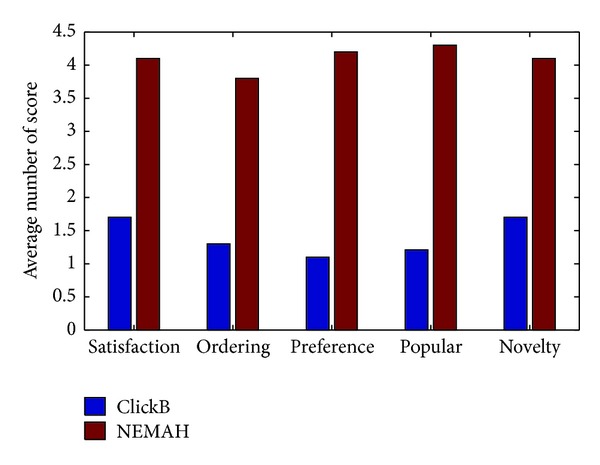
User study on different metrics.

In order to get the other evaluated metrics to verify our proposed news recommendation system, we develop a prototype system of our proposed NEMAH and design a questionnaire which includes the following questions: (1) satisfaction of news content; (2) ordering of the recommendation list; (3) preference of the news subclasses; (4) popularity of news article; and (5) novelty of the recommendation list. For each question, we define 5 indexes for selection, where 1:* So Bad*, 2:* Not Very Well*, 3:* Average*, 4:* Good but Needs to Improve*, and 5:* Excellent*. We crawl news articles of the latest three days from several famous news websites as a candidate set for recommendation. At last, we hire 50 volunteers who are required to have microblog account in SINA website to help us complete the questionnaire. We send them the same questionnaire with different recommendation lists every week for three times. The average result of this user study is shown in Figure 11. From the result of user study, we can see that NEMAH can satisfy the requirements represented by our questions of most of people.

## 9. Conclusion

In this paper, we proposed NEMAH system architecture to tackle the personalized news recommendation based on microblog and subclass popularity prediction. We explore the intrarelations among microblog content and news items and, considering the subclass popularity factor, similarity among users, place, and key person factors synthetically. Our system supports effective classification and subclass clustering on newly published news articles along with a few of history corpus. High quality of classification and clustering can construct a better data structure for recommending. Experimental results compared with some state-of-the-art algorithms have demonstrated the better performance of NEMAH. Besides, our work in Sections 4 and 5 can be utilized for automatic module layout and channel ranking.

For future work, to process mass network news articles, we plan to deploy some components (e.g., classification, clustering, and subclass popularity analysis) onto the Map-Reduce framework on our distributed system. Moreover, we also plan to integrate the subclass popularity prediction module into our news search engine due to the effectiveness in our work. Another remarkable point is the interest evolution of users (e.g., time, place, and other factors), which is able to provide insights on the exploration of news reading behaviors.

## Figures and Tables

**Table 1:** Name of each class.

ID	Class name
1	Political
2	Law, Justice
3	External Relations, International Relations
4	Military
5	Social, Labor, Disaster
11	Economy
12	Finance, Banking
13	Infrastructure, Construction, Real estate
14	Agriculture, Rural areas
15	Mining, Industrial
16	Energy, Water, Conservancy
17	Information industry
18	Transport, Postal services, Logistics
19	Commerce, Foreign trade, Customs
21	Services, Tourism
22	Environmental, Meteorological
31	Education
33	Science and Technology
35	Culture, Recreation, and Leisure
36	Literature, Art
37	Media Industry
38	Medicine, Health
39	Sports

**Table 2:** Recommendation Micro-F1 (Top@30) of different time periods for different classification based systems.

Range (Y.M)	#	NB	Cheng	Z.Guo	NEMAH
09.08-09.08	4,239	0.204	0.242	0.270	0.351
09.10–09.12	37,910	0.206	0.254	0.268	0.364
10.01–10.06	75,047	0.227	0.289	0.297	0.403
10.07–11.07	151,995	0.198	0.271	0.274	0.371
09.08–12.08	280,737	0.210	0.273	0.284	**0.383**

Remark: ^#^denotes the number of news articles. Time range 09.08 denotes August, 2009.

**Table 3:** Diversity evaluation on different recommendation lists.

Methods	T@10	T@20	T@30
Goo	0.5104	0.4320	0.1215
ClickB	0.5231	0.4457	0.1587
Bilinear	0.5024	0.3547	0.1478
Bandit	0.6112	0.3874	0.2674
SCENE	0.6821	0.5747	0.5687
NEMAH	**0.7425**	**0.6941**	**0.6637**

*Remark. *T at *n*-recommended result with top-*n*.
